# Modulation of Legume Defense Signaling Pathways by Native and Non-native Pea Aphid Clones

**DOI:** 10.3389/fpls.2016.01872

**Published:** 2016-12-15

**Authors:** Carlos Sanchez-Arcos, Michael Reichelt, Jonathan Gershenzon, Grit Kunert

**Affiliations:** Department of Biochemistry, Max Planck Institute for Chemical EcologyJena, Germany

**Keywords:** *Acyrthosiphon pisum*, pea aphid host races, plant hormones, salicylic acid, jasmonates, abscisic acid

## Abstract

The pea aphid (*Acyrthosiphon pisum*) is a complex of at least 15 genetically different host races that are native to specific legume plants, but can all develop on the universal host plant *Vicia faba*. Despite much research, it is still unclear why pea aphid host races (biotypes) are able to colonize their native hosts while other host races are not. All aphids penetrate the plant and salivate into plant cells when they test plant suitability. Thus plants might react differently to the various pea aphid host races. To find out whether legume species vary in their defense responses to different pea aphid host races, we measured the amounts of salicylic acid (SA), the jasmonic acid-isoleucine conjugate (JA-Ile), other jasmonate precursors and derivatives, and abscisic acid (ABA) in four different species (*Medicago sativa*, *Trifolium pratense*, *Pisum sativum*, *V. faba*) after infestation by native and non-native pea aphid clones of various host races. Additionally, we assessed the performance of the clones on the four plant species. On *M. sativa* and *T. pratense*, non-native clones that were barely able to survive or reproduce, triggered a strong SA and JA-Ile response, whereas infestation with native clones led to lower levels of both phytohormones. On *P. sativum*, non-native clones, which survived or reproduced to a certain extent, induced fluctuating SA and JA-Ile levels, whereas the native clone triggered only a weak SA and JA-Ile response. On the universal host *V. faba* all aphid clones triggered only low SA levels initially, but induced clone-specific patterns of SA and JA-Ile later on. The levels of the active JA-Ile conjugate and of the other JA-pathway metabolites measured showed in many cases similar patterns, suggesting that the reduction in JA signaling was due to an effect upstream of OPDA. ABA levels were downregulated in all aphid clone-plant combinations and were therefore probably not decisive factors for aphid-plant compatibility. Our results suggest that *A. pisum* clones manipulate plant-defense signaling to their own advantage, and perform better on their native hosts due to their ability to modulate the SA- and JA-defense signaling pathways.

## Introduction

More than 5000 aphid species are known today (Blackman and Eastop, 2000), with at least part of the diversity due to sympatric speciation initiated by individuals that switched to new host plants (Diehl and Bush, 1984; Dres and Mallet, 2002). When aphids switch to new plants they may be confronted with new defense mechanisms (Goggin, 2007; Smith and Boyko, 2007; Howe and Jander, 2008; Wu and Baldwin, 2010) and so may be unable to establish a compatible interaction. In most cases plants will recognize new aphid invaders on the basis of herbivore-associated molecular patterns (HAMPs) that lead to HAMP-triggered immunity (Hogenhout and Bos, 2011; Kaloshian and Walling, 2016). Among the major aphid HAMPs studied are salivary proteins, such as a 3–10 kDa protein from *Myzus persicae* that can induce a defense response in *Arabidopsis thaliana* (De Vos and Jander, 2009). Several *M. persicae* salivary HAMPs have been shown to be detrimental to aphids and reduced their fecundity on *A. thaliana* and *Nicotiana tabacum* (Bos et al., 2010; Elzinga et al., 2014) presumably because of the defense reactions they trigger. For example, HAMPs induce an influx of Ca^2+^ ions, an important second messenger in signaling actions (Wu and Baldwin, 2010). Ca^2+^ ions are associated with the production of reactive oxygen species (ROS) and other defense responses (Chen et al., 1993; Mai et al., 2013; Herrera-Vasquez et al., 2015).

The best studied defense reaction in plants is the formation of phytohormones involved in signal transduction pathways (Mauch-Mani and Mauch, 2005; Pieterse et al., 2009, 2012; Cao et al., 2011; Morkunas et al., 2011; Denance et al., 2013; Wasternack and Hause, 2013; Caarls et al., 2015), among which salicylic acid (SA) and jasmonic acid-isoleucine (JA-Ile) are the two main defense-related compounds. While the SA-defense pathway has mainly been associated with the response against biotrophic pathogens, the jasmonic acid (JA-) defense pathway, mainly activated after wounding (Howe, 2004), affects herbivorous insects and necrotrophic pathogens (Pieterse et al., 2012). Both defense pathways are, however, strongly interconnected (De Vos et al., 2005; Beckers and Spoel, 2006; Koornneef and Pieterse, 2008; Pieterse et al., 2009; Gimenez-Ibanez and Solano, 2013; Caarls et al., 2015), and it is reported that SA can negatively affect JA signaling downstream of the SCF^COI1^-JAZ complex (Koornneef et al., 2008; Zhang et al., 2009, 2013; Van Der Does et al., 2013), and that JA can suppress the SA-defense pathway (Brooks et al., 2005; Nomura et al., 2005). Synergistic interactions between SA and JA signaling have also been detected (Schenk et al., 2000; Mur et al., 2006). Additionally the timing and the sequence of SA and JA signaling initiation (Koornneef et al., 2008; Leon-Reyes et al., 2010) as well as the levels of phytohormones seem to be important for certain defense responses (Mur et al., 2006). Other phytohormones like abscisic acid (ABA) play an important role in fine tuning the defense reponse of the plants and interfere with JA and SA signaling (Mauch-Mani and Mauch, 2005; Ton et al., 2009; Cutler et al., 2010; Cao et al., 2011; Morkunas et al., 2011; Denance et al., 2013). Initially, ABA promotes early defense responses, closing stomata and stimulating callose deposition, which blocks the intrusion of the pathogen into plant tissue. In late responses, ABA interacts with other defense pathways inhibiting the SA-dependent responses or modulating the JA-dependent pathway (Yasuda et al., 2008; Ton et al., 2009; Pieterse et al., 2012; Finkelstein, 2013). Much is still to be learned about the regulation of hormonal cross talk. Nonetheless, it is assumed that these mechanisms provide plants with an adaptable system capable of tuning defense responses to different classes of attackers (Pieterse et al., 2012) and resulting in the formation of toxic or deterrent defense compounds that prevent the colonization of the plant.

Aphids employ a range of strategies to overcome plant defense (Walling, 2008; Giordanengo et al., 2010; Kamphuis et al., 2013; Will et al., 2013; Jaouannet et al., 2014). They may detoxify defense compounds, induce nutrient sinks or sequester calcium to block phloem sealing. However, many of the effector proteins in aphid saliva may hinder activation of plant defenses and so may decrease phytohormone signaling. For example, Mp55, an effector molecule from *M. persicae* suppressed the formation of three defense compounds in *A. thaliana*: 4-methoxyindol-3-ylmethyl glucosinolate, callose and hydrogen peroxide (Elzinga et al., 2014). A structural protein of the stylet sheath, important for sealing the stylet penetration site, might prevent the influx of Ca^2+^ ions and the activation of Ca^2+^-dependent defense signaling machinery (Abdellatef et al., 2015; Furch et al., 2015). Calcium-binding proteins in aphid saliva seem to have the same effect (Will et al., 2007). In other cases, the mode of action of salivary effectors is not known. However, effector proteins like Armet and C002 from *A. pisum* (Mutti et al., 2006, 2008; Wang et al., 2015), Me10 and Me23 from the potato aphid *Macrosiphum euphorbiae*, and PIntO1 and PIntO2 from the green peach aphid *M. persicae* enhance performance on the respective host plants (Pitino and Hogenhout, 2013), and silencing of the encoding genes by RNAi reduced aphid fecundity (Mutti et al., 2006, 2008; Bos et al., 2010; Pitino et al., 2011). These proteins may also interfere with defense-signaling pathways and so alter phytohormone levels. Thus the measurement of phytohormone levels after aphid infestation may provide excellent indications about whether these insects trigger or block defense signaling on different host plants.

One of the best studied aphid species is the pea aphid *Acyrthosiphon pisum* whose genome was the first to be completely sequenced among hemipterans (The International Aphid Genomics Consortium, 2010). The pea aphid is a legume specialist feeding on crops like lentil, bean, pea, alfalfa, and clover, as well as wild legume species. About 6200 years ago it underwent a rapid diversification, which led to the development of at least 15 different sympatric host races or biotypes specialized on certain host plants (Ferrari et al., 2006, 2008; Peccoud et al., 2009a,b, 2015). A pea aphid host race performs best on its native host plant, and has a reduced fitness or cannot survive at all on other legume species. However, all pea aphid host races can perform well, sometimes best on *Vicia faba*, the universal host plant for all pea aphid biotypes characterized to date. The mechanisms that are involved in this host specialization are mostly unknown. There were attempts to find the genomic regions associated with plant adaptation of pea aphid host races (Hawthorne and Via, 2001; Jaquiery et al., 2012; Simon et al., 2015). A genome-wide study of pea aphid host races was conducted and a few loci encoding salivary proteins were identified in regions under putative divergent selection (Jaquiery et al., 2012). Investigation of feeding behavior revealed that regardless of whether they are on their native host plant or another legume species, pea aphids start to penetrate the plant and to pierce and salivate into plant cells (Schwarzkopf et al., 2013). In order to find out what is salivated into the plant, transcriptomic analysis of salivary glands was conducted and around 600 pea aphid salivary genes were described (Carolan et al., 2011). In addition, proteins were identified by proteomic analysis of saliva (collected from artificial diet fed by aphids) or salivary glands (Carolan et al., 2009, 2011; Vandermoten et al., 2014). These salivary proteins may suppress plant-defense responses in native host plants (Will et al., 2007; Mutti et al., 2008; Pitino and Hogenhout, 2013) or trigger defense reactions in non-host plants (Li et al., 2006; Gao et al., 2008; Hogenhout and Bos, 2011). To investigate these roles, it would be useful to determine how phytohormone levels differ among various host race-host species combinations.

The pea aphid complex has become a model system for asking questions about the origin and maintenance of feeding specialization in insect herbivores. To find out why host races can perform well on their native or the universal host plant while they are not able to colonize other plants, an important step would be to measure the defense phytohormone levels to determine whether defenses are being activated or not. The detection of differences in phytohormone levels induced by native vs. non-native host races would favor the hypothesis that native aphid races are able to manipulate plant-defense activation processes for their own benefit. So far, there is just one study investigating the phytohormone response of a native host plant (*Pisum sativum*) to pea aphid infestation (Mai et al., 2014). This study however, concentrated on changes due to aphid numbers and only used an aphid clone that was native to *P. sativum*. Thus information about how pea aphid host plants react to non-native pea aphid host races is still lacking. Therefore, in this study we investigated the phytohormone response of three native host plants of the pea aphid, *Medicago sativa*, *P. sativum*, *Trifolium pratense*, and the universal host *V. faba* over a 4-day time course after infestation with native and non-native aphid clones. We analyzed levels of the JA-Ile conjugate, SA, and ABA, and also quantified several other jasmonate metabolites to explore how aphids might manipulate hormone signaling by interfering with specific biosynthetic steps. In addition, we determined the perfomance of native and non-native aphid host races on each plant species. Although data are available in the literature on pea aphid reproduction on different hosts, this information is for plants of different ages and varieties and from different growing conditions that what was used here, and did not assess the survival and growth of adult aphids.

## Materials and Methods

### Plant Material

Four legume plant species: *M. sativa* cultivar (cv.) ‘Giulia’ (alfalfa), *T. pratense* cv. ‘Dajana’ (red clover), *P. sativum* cv. ‘Baccara’ (pea), and *V. faba* cv. ‘The Sutton’ (broad bean), were grown in 7-cm diameter plastic pots with a standardized soil mixture (7:20 mixture of Klasmann Tonsubstrat and Klasmann Kultursubstrat TS1, Klasmann-Deilmann GmbH, Geeste, Germany) in climate chambers maintained at 20°C, 70 ± 10% relative humidity, and 16-h light/8-h dark photoperiod. *M. sativa* and *T. pratense* were grown three plants per pot in order to get enough plant material for phytohormone analyses (approximately 10 and 6 leaves per pot, respectively), while *P. sativum* and *V. faba* were grown individually (approximately 4 leaves per pot for each species). *M. sativa* and *T. pratense* plants were used in experiments 20 days after sowing and *P. sativum* and *V. faba* 10 days after sowing.

### Aphids

Three pea aphid (*A. pisum* Harris) clones, each representing one pea aphid host race, were used in the experiments: the clone L84 representing the *Medicago* race (here called MR), the clone T3-8V1 representing the *Trifolium* race (TR), and the clone Colmar representing the *Pisum* race (PR). Aphids were initially collected from their native host plants *T. pratense*, *M. sativa*, and *P. sativum*, respectively, and genotypically assigned to their respective host race [for detailed information see Supplementary Table S1 in Peccoud et al. (2009a)]. All aphids were reared on 4-week-old broad bean plants. To synchronize the age of the aphids for the experiments, five apterous female adults were placed on a broad bean plant and were allowed to reproduce for 48 h. The nymphs were then transferred to new plants and maintained for 9 days until they reached the adult age. Several serial transfers of nymphs were done until the desired number of synchronized young adult aphids was obtained. To avoid escape of aphids, all aphid containing plants were covered with air permeable cellophane bags (18.8 cm × 39 cm, Armin Zeller, Nachf. Schütz & Co, Langenthal, Switzerland), and placed in a climate chamber under the conditions described above.

### Experimental Design

To determine the performance of the three different pea aphid clones of various host races, each plant species was separately infested with each pea aphid clone resulting in 12 plant species–aphid clone combinations. To evaluate the development of the different pea aphid clones over time, plants were infested with 20 adult, apterous aphids, and performance parameters were measured 24, 48, 72, and 96 h after aphid infestation and at the start of the experiment. Survival and mean weight of adult aphids (weight of all alive adult aphids on a plant divided by the number of surviving adult aphids), and the weight of all offspring per plant were measured as performance parameters. To keep the aphids as undisturbed as possible (and to duplicate the setup used in the phytohormone experiment described below), different sets of plants and aphids were used at each time point. For this performance experiment, five replicates were used.

To evaluate the response of the plant species toward infestation with the different pea aphid clones, phytohormone levels were investigated. The experimental setup was the same as for the performance experiment with 12 plant species – aphid clone combinations sampled at four-time points. Additionally plants without aphids served as controls. Ten replicates were employed.

All experimental plants, including aphid-free control plants, were covered with air permeable cellophane bags and were placed in a climate chamber under conditions as described above.

### Plant Material Sampling and Extraction

For plant sampling, the aphids were removed from the plants using a paintbrush. As a control for possible induction of phytohormones due to contact with the paintbrush, control plants were brushed in the same way as aphid-infested plants. Above-ground parts of the plant seedlings were harvested and rapidly frozen in liquid nitrogen. Frozen samples were stored overnight in 2-ml Eppendorf tubes at -80°C and then freeze-dried for 48 h. Dried plant material was homogenized into a fine powder by adding three stainless steel beads (3 mm Ø) in each tube and vigorously shaking for four min in a paint shaker (Skandex shaker SO-10 m, Fast and Fluid Management, Sassenheim, The Netherlands). Portions (10 mg) of dried plant material were extracted with 1 ml ice-cold extraction solution containing 80% methanol acidified with 0.1% formic acid with deuterated or ^13^C-labeled phytohormones as internal standards, (40 ng ml^-1^ of jasmonic acid-d_6_, SA-d_4_, and ABA-d_6_, and 8 ng ml^-1^ of jasmonic acid-^13^C_6_-isoleucine conjugate). Samples were immediately vortexed for 10 s and continuously sonicated in a water bath at room temperature (20°C) for 15 min at maximum frequency (35 kHz). After centrifugation (10 min at 4,500 *g* and -10°C), supernatants were filtered using 0.45 mm PTFE AcroPrep^TM^ 96-well filtration plates (Pall Corporation, Port Washington, NY, USA) and a vacuum filtration unit. All filtered plant extracts were stored at -80°C until LC-MS/MS analysis.

### Quantification of Phytohormones by LC-MS/MS

Chromatographic separation of phytohormones was performed on an Agilent 1260 HPLC system (Agilent Technologies, Santa Clara, CA, USA). Separation was achieved on a Zorbax Eclipse XDB-C18 column (50 mm × 4.6 mm, 1.8 μm, Agilent). Formic acid (0.05%) in water and acetonitrile were employed as mobile phases A and B, respectively. The elution profile was: 0–0.5 min, 10% B; 0.5–4.0 min, 10–90% B; 4.0–4.02 min, 90–100% B; 4.02–4.50 min, 100% B, 4.50–4.51 min 100–10% B, and 4.51–7.00, 10% B. The mobile phase flow rate was 1.1 ml/min. The column temperature was maintained at 25°C. An API 5000 tandem mass spectrometer (Applied Biosystems, Foster City, CA, USA) equipped with a Turbospray ion source was operated in negative ionization mode. The instrument parameters were optimized by infusion experiments with pure standards, where available. The ion spray voltage was maintained at -4500 eV. The turbo gas temperature was set at 700°C. Nebulizing gas was set at 60 psi, curtain gas at 25 psi, the heating gas at 60 psi and collision gas at 7 psi. Multiple reaction monitoring (MRM) was used to monitor analyte parent ion → product ion fragmentations as follows: *m*/*z* 136.9→93.0 (collision energy (CE) -22 V; declustering potential (DP) -35 V) for SA; *m*/*z* 140.9→97.0 (CE -22 V; DP -35 V) for SA-d_4_; *m*/*z* 290.9→165.1 (CE -24 V; DP -45 V) for 12-oxo phytodienoic acid (OPDA); *m*/*z* 209.1→59.0 (CE -24 V; DP -35 V) for JA; *m*/*z* 215.1→59.0 (CE -24 V; DP -35 V) for JA-d_6_; *m*/*z* 225.1→59 (CE -24 V; DP -35 V) for the two hydroxyjasmonic acid isomers (here designated OH-JA1 and OH-JA2, respectively); *m*/*z* 322.2→130.1 (CE -30 V; DP -50 V) for JA-Ile; *m*/*z* 328.2→136.1 (CE -30 V; DP -50 V) for JA-^13^C_6_-Ile; *m*/*z* 338.1→130.1 (CE -30 V; DP -50 V) for 12-OH-JA-Ile; *m*/*z* 352.1→130.1 (CE -30 V; DP -50 V) for 12-carboxyjasmonic acid-isoleucine conjugate (12-COOH-JA-Ile); *m*/*z* 263.0→153.2 (CE -22 V; DP -35 V) for ABA; *m*/*z* 269.0→159.2 (CE -22 V; DP -35 V) for ABA-d_6_. The hydroxyjasmonic acids include the 11- and 12-hydroxy derivatives (Miersch et al., 2008; Stitz et al., 2011), but we were unable to distinguish between them. Both Q1 and Q3 quadrupoles were maintained at unit resolution. Analyst 1.6 software (Applied Biosystems) was used for data acquisition and processing. Linearity in ionization efficiencies was verified by analyzing dilution series of standard mixtures. Phytohormones were quantified relative to the signal of their corresponding internal standard. For quantification of OPDA and OH-JA, the internal standard JA-d_6_ was used applying experimentally determined response factors of 0.5 and 1.0, respectively. These response factors were determined by analyzing a mixture of OPDA and OH-JA [both kindly provided by W. Boland, MPI for Chemical Ecology, Jena, Germany; synthesized as described in Nakamura et al. (2011) and Shabab et al. (2014)] and JA-d_6_ all at the same concentration. For OH-JA-Ile and COOH-JA-Ile quantification, JA-^13^C_6_-Ile was used as internal standard applying a response factor of 1.0 in both cases. The response factor for OH-JA-Ile was determined by analyzing a mixture of OH-JA-Ile [kindly provided by W. Boland, MPI for Chemical Ecology, Jena, Germany; synthesized as described in Jimenez-Aleman et al. (2015)] and JA-^13^C_6_-Ile at the same concentration. The response factor for COOH-JA-Ile was assumed to be similar. All metabolite levels are expressed in nanograms per gram dry weight (ng g^-1^ DW).

### Chemicals

The sources of the phytohormone standards were jasmonic acid-d_6_ (HPC Standards GmbH, Cunnersdorf, Germany), SA-d_4_ (Sigma–Aldrich), ABA-d_6_ (Santa Cruz Biotechnology, Dallas, TX, USA), and jasmonic acid-^13^C_6_-isoleucine conjugate [synthesized as described by Kramell et al. (1988) using ^13^C_6_-Ile (Sigma–Aldrich)].

The sources of the solvents used for the phytohormone extraction were methanol (LiChrosolv^®^, LC-MS grade, Merck KGaA, Germany), acetonitrile (LC-MS grade, VWR Chemicals, USA), and formic acid (LC-MS grade, Fisher Scientific, Belgium).

### Statistical Analysis

All data were analyzed with R version 3.2.0 (R Development Core Team, 2015).

The percentage of surviving adults was analyzed using binomial generalized linear models (glm) with time after aphid infestation as continuous and aphid clone as categorical explanatory variables. In cases of overdispersion, standard errors were corrected using quasi-glm models. *P*-values for explanatory variables were obtained by deleting explanatory variables one after another and comparison of the most complex model with the simpler model (Zuur et al., 2009).

To make the progression of aphid weight over time comparable between the different aphid clones, the weight of surviving adult aphids is given as a percentage of the weight at the start of the experiment, which was set as 100%. These data were analyzed using a two-way ANOVA with the time points and aphid clones as categorical explanatory variables. Models were simplified by deleting non-significant variables (Crawley, 2013). To determine differences between factor levels, pairwise *t*-tests were performed and corrected for the false discovery rate. In cases where variances were unequal, the generalized least squares method [gls from the nlme library (Pinheiro et al., 2015)] was used. First, the optimal variance structure was determined by comparing models with different variance structures and choosing the one with the smallest AIC (Akaike information criterion). Models with this variance structure were used to determine the influence of explanatory variables by subsequent removal of explanatory variables from the model and comparison of the simpler with the more complex model with a likelihood ratio test (Zuur et al., 2009). Differences between factor levels were determined by factor level reduction (Crawley, 2013).

The influence of the aphid clone and time on the offspring biomass was investigated with a two-way ANOVA. To achieve homogeneity of variances, biomass data were square root transformed. Differences between factor levels were examined by pairwise *t*-tests corrected for false discovery rate.

The influence of aphid clone and duration of aphid infestation (both used as categorical explanatory variables) on the phytohormone levels was investigated using the generalized least squares method [gls from the nlme library (Pinheiro et al., 2015)] to account for the variance heterogeneity of the residuals. The varIdent variance structure was used. Whether the different variance of aphid clones, the duration of aphid infestation or the combination of both factors should be incorporated into the model, was determined by comparing models with different variance structures with a likelihood ratio test and choosing the model with the smallest AIC. The influence (*p*-values) of the explanatory variables was determined as explained above in the analysis of adult weight.

## Results

### Aphid Host Race Clones Performed Much Better on Their Native Host Plants

To evaluate the performance of pea aphid clones of various host races on different plants over time, we determined the survival and weight of adult aphids, and the total weight of aphid offspring.

**FIGURE 1:**
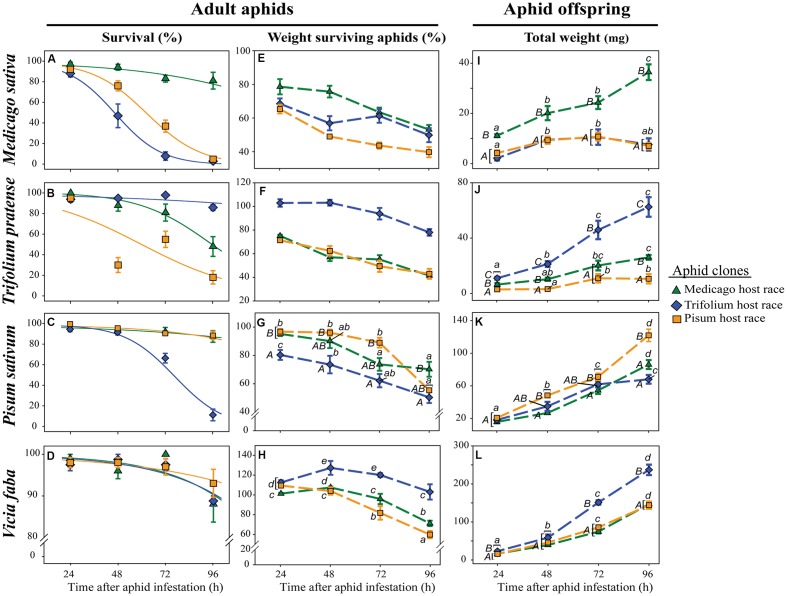
**Performance of pea aphid clones of different host races on native and non-native legume species**. Survival of adult aphids **(A–D)**, mean weight of surviving adult aphids **(E–H)**, and total weight of offspring **(I–L)** are depicted for three aphid clones tested on *M. sativa*, *T. pratense*, *P. sativum*, and *V. faba* plants and measured 24, 48, 72, and 96 h after aphid infestation. The aphid clones are from the *Medicago*, *Trifolium*, and *Pisum* host races. Symbols represent means ± SE. Statistical values are given in tables 1 **(A–D)**, 2 **(E–H)**, and 3 **(I–L)**. In cases where a significant influence of the aphid clone on the weight of the surviving adults or the total weight of the offspring was dependent on the time after aphid infestation (time × race interaction), *post hoc* tests or similar methods were used to reveal differences between aphid clones at different time points. Different letters indicate significant differences (*P* ≤ 0.05). Upper case letters in **(G,I–L)** indicate significant differences between aphid clones within a certain time point, while lower case letters indicate significant differences between different time points within one aphid clone. **(A–D)** Solid lines in the survival graphs are the fitted curves from the generalized linear model (glm). **(E–H)** The mean weight of surviving adult aphids is given as percentage of the weight at the start of the experiment which was set as 100%.

The survival of all aphid clones on all host plants decreased over time. The strength of the decrease was, however, dependent on the plant – aphid clone combination. On their respective native host plant or the universal host plant *V. faba*, more than 80% of the aphids survived for 4 days (96 h). This survival was significantly better than the survival of non-native clones (**Figures 1A–C**). On *M. sativa* hardly any (<2) of the non-native aphids survived for 96 h (**Figure 1A**). On *T. pratense* on average only 18% of the non-native *Pisum* clone (PR) survived, whereas about 48% of the non-native *Medicago* clone (MR) survived (**Figure 1B**). The only exception from this general pattern was found for aphids on *P. sativum*. There the non-native MR survived as well as the native PR, and only the non-native *Trifolium* clone (TR) showed a strongly reduced survival (**Figure 1C**). On the universal host plant *V. faba* all aphid clones survived equally well (**Figure 1D**; **Table 1**).

**Table 1:** Statistical values for the analysis of the survival of adult aphids on different legume species according to aphid clone, time of aphid infestation, and the interaction between aphid clone and time of aphid infestation.

Plant species	Statistical test used	Factor	F/*Deviance*	*P*-value
*M. sativa*	glm/quasibinomial	Interaction	9.393	**<0.001**
		Clone	61.897	**<0.001**
		Time	39.620	**<0.001**
*T. pratense*	glm/quasibinomial	Interaction	2.201	0.121
		Clone	32.077	**<0.001**
		Time	17.905	**<0.001**
*P. sativum*	glm/quasibinomial	Interaction	9.402	**<0.001**
		Clone	29.848	**<0.001**
		Time	36.724	**<0.001**
*V. faba*	glm/binomial	Interaction	*-0.774*	0.679
		Clone	*-0.457*	0.796
		Time	*-21.990*	**<0.001**

*Significant *P*-values are given in bold. Depending which statistical test was used F-values or Deviance are given. Deviance values are given in italics*.

Surviving adult aphids on all plants lost weight significantly during the experiment (**Figures 1E–H**; **Table 2**). In general, the aphid clones on their native host plants lost significantly less weight than non-native clones. This pattern was most pronounced on *T. pratense* plants, where the native TR lost about 20% of its initial weight over the course of the experiment, whereas both non-native clones (MR and PR) lost about 60% of their original weight (**Figure 1F**). Also, on *M. sativa* both non-native clones were significantly lighter than the native MR (**Figure 1E**). On *P. sativum*, the non-native TR lost significantly more weight than the non-native MR and the native PR (**Figure 1G**). In contrast, on the universal host *V. faba*, aphids of all clones either kept their initial weight for the first 2–3 days or even gained weight. Only after this time did they start to lose weight (**Figure 1H**).

**Table 2:** Statistical values for the analysis of the weight of surviving adult aphids on different legume species according to aphid clone, time of aphid infestation, and the interaction between aphid clone and time of aphid infestation.

Plant species	Statistical test used	Factor	F/*L-ratio*	*P*-value
*M. sativa*	ANOVA	Interaction	2.105	0.072
		Clone	30.790	**<0.001**
		Time	24.190	**<0.001**
*T. pratense*	ANOVA	Interaction	1.722	0.137
		Clone	152.140	**<0.001**
		Time	36.520	**<0.001**
*P. sativum*	ANOVA	Interaction	2.841	**0.019**
		Clone	24.307	**<0.001**
		Time	37.734	**<0.001**
*V. faba*	gls/varIdent error structure for each time-clone combination	Interaction Clone Time	*35.768 24.540 28.487*	**<0.001** **<0.001** **<0.001**

*Significant *P*-values are given in bold. Depending which statistical test was used F-values or Likelihood ratios are given. Likelihood ratios are given in italics*.

The highest amount of aphid offspring produced during the experiment came from aphid clones on their native host plants. The total weight of these offspring increased significantly over time and was always significantly higher than the weight of offspring from non-native aphid clones (**Figures 1I–K**; **Table 3**). On *M. sativa* non-native aphids produced only a few offspring. After 96 h the total weight of their offspring added up to only one-fifth of that of native aphids (**Figure 1I**). The same was observed for the non-native PR on *T. pratense*, but there the non-native MR could produce about 40% the weight of offspring produced by the native TR (**Figure 1J**). On *P. sativum*, the weight of offspring over time increased for all aphid clones but with a significantly stronger increase for the native PR (**Figure 1K**). A significant increase in offspring weight for all aphid clones was also found on the universal host *V. faba*. On this plant, the offspring weight was always highest compared to offspring weight on other plants, but also differed between aphid clones. TR produced a significantly higher mass of offspring than the other clones. (**Figure 1L**).

**Table 3:** Statistical values for the analysis of the total weight of offspring produced on different legume species according to aphid clone, time of aphid infestation, and the interaction between aphid clone and time of aphid infestation.

Plant species	Transformation	Factor	*F*-value	*P*-value
*M. sativa*	sqrt	Interaction	3.655	**0.005**
		Clone	67.914	**<0.001**
		Time	19.396	**<0.001**
*T. pratense*	sqrt	Interaction	3.936	**0.003**
		Clone	84.247	**<0.001**
		Time	42.997	**<0.001**
*P. sativum*	sqrt	Interaction	5.113	**<0.001**
		Clone	28.904	**<0.001**
		Time	216.371	**<0.001**
*V. faba*	sqrt	Interaction	7.479	**<0.001**
		Clone	66.321	**<0.001**
		Time	481.858	**<0.001**

*Significant *P*-values are given in bold*.

### Clones of Native Host Races Induced Lower Levels of SA and JA-Ile Than Clones of Non-native Races

To determine how the pea aphid clones of the various host races affected the defense response of the different plant species, we measured the amounts of three plant hormones known to be involved in defense signaling, SA, JA-Ile, and ABA, in each plant species separately infested with each of the aphid clones and in uninfested control plants.

**FIGURE 2:**
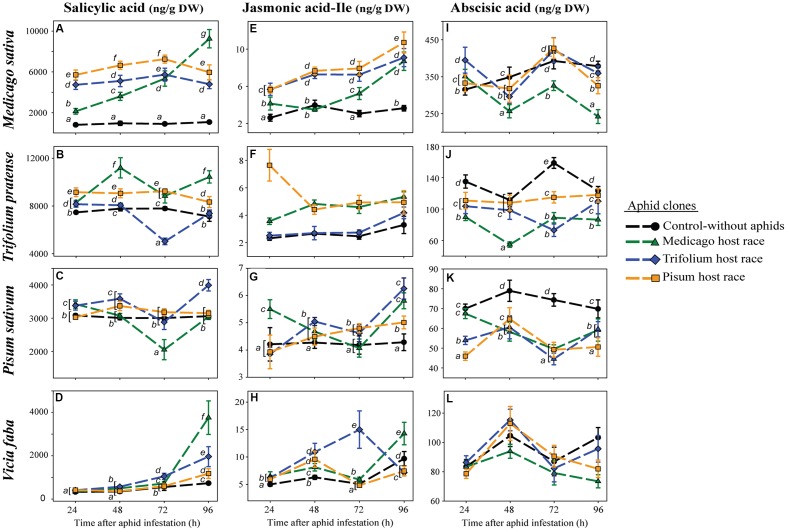
**Levels of salicylic acid **(A–D)**,** jasmonic acid-isoleucine **(E–H)** and abscisic acid **(I–L)** in legume plants after infestation with pea aphid clones of different host races. Symbols represent means ± SE. Statistical values are presented in **Table 4**. In cases where a significant influence of the aphid clone on the phytohormone level was dependent on the time after aphid infestation (interaction), significant differences (*P* ≤ 0.05) between aphid clones at different time points are indicated by different letters.

Although SA levels in uninfested control plants changed only slightly over time, large changes were occasionally observed in aphid-infested plants (**Figures 2A–D**; **Table 4**). These changes occurred in an aphid clone-specific manner. In *T. pratense*, the SA levels after infestation with the non-native clones were always significantly higher than the ones observed after infestation with the native clone and the ones occurring in uninfested control plants. Depending on the time point, SA levels in plants infested with the native aphid clone were higher, equal or lower than the levels in uninfested control plants (**Figure 2B**). In *M. sativa*, all aphid clones elicited a significant increase in SA levels. As in *T. pratense* this increase was significantly higher in plants infested with non-native aphid clones than in plants with the native aphid clone for the first 72 h after aphid infestation. However, after this time the SA levels in plants with the non-native aphid clones decreased whereas the levels in plants infested with the native MR clone increased to significantly higher levels (**Figure 2A**).

**Table 4:** Statistical values for the analysis of phytohormone levels in different legume species according to aphid clone, time of aphid infestation, and the interaction between aphid clone and time of aphid infestation.

Phytohormone	Factor	*Medicago sativa*			*Trifolium pratense*			*Pisum sativum*			*Vicia faba*		
		Variance	*L*-ratio	*P*-value	Variance	*L*-ratio	*P*-value	Variance	*L*-ratio	*P*-value	Variance	*L*-ratio	*P*-value
SA	Interaction	Treat	39.153	**<0.001**	Treat	50.454	**<0.001**	Treat	36.765	**<0.001**	Treat	27.395	**0.001**
	Time		7.336	0.062		8.873	**0.031**		1.682	0.641		47.924	**<0.001**
	Clone		222.283	**<0.001**		52.913	**<0.001**		15.691	**0.001**		7.499	0.058
JA	Interaction	Treat	25.652	**0.002**	Treat	46.036	**<0.001**	Treat	59.924	**<0.001**	Treat	46.836	**0.001**
	Time		35.843	**<0.001**		2.629	0.452		17.347	**<0.001**		41.140	**<0.001**
	Clone		58.841	**<0.001**		84.842	**<0.001**		11.623	**0.009**		10.147	**0.017**
ABA	Interaction	Treat	29.659	**<0.001**	Race	25.066	**0.003**	Time	31.306	**<0.001**	Time	12.635	0.180
	Time		24.361	**<0.001**		21.067	**<0.001**		9.962	**0.019**		22.568	**<0.001**
	Clone		10.416	**0.015**		61.095	**<0.001**		63.165	**<0.001**		5.243	0.155
OPDA	Interaction	Treat	83.702	**<0.001**	Treat	40.459	**<0.001**	Treat	33.652	**<0.001**	Treat	61.859	**<0.001**
	Time		41.687	**<0.001**		3.666	0.300		20.965	**<0.001**		58.519	**<0.001**
	Clone		75.721	**<0.001**		51.441	**<0.001**		16.154	**0.001**		10.430	**0.015**
JA-Ile	Interaction	Treat	36.223	**<0.001**	Treat	15.788	0.071	Time	23.719	**0.005**	Treat	26.854	**0.002**
	Time		17.566	**<0.001**		19.783	**<0.001**		17.909	**<0.001**		34.264	**<0.001**
	Clone		99.135	**<0.001**		78.228	**<0.001**		11.373	0.251		8.941	**0.030**
OH-JA1	Interaction	Treat	29.956	**<0.001**							Time	37.378	**<0.001**
	Time		45.957	**<0.001**	Not detectable			Not detectable				13.691	**0.003**
	Clone		49.960	**<0.001**								11.000	**0.012**
OH-JA2	Interaction	Treat	76.052	**<0.001**	Treat	19.502	**0.021**				Treat	37.372	**<0.001**
	Time		7.005	0.072		26.260	**<0.001**	Not detectable				34.891	**<0.001**
	Clone		117.077	**<0.001**		91.853	**<0.001**					1.158	0.763
OH-JA-Ile	Interaction	Treat	82.928	**<0.001**	Race	10.144	0.339				Treat	13.692	0.134
	Time		12.537	**0.006**		2.353	0.502	Not detectable				28.695	**<0.001**
	Clone		23.852	**<0.001**		53.008	**<0.001**					9.006	**0.029**
COOH-JA-Ile	Interaction	Treat	29.033	**<0.001**	Treat	27.277	**0.001**						
	Time		4.712	0.194		7.143	0.068	Not detectable			Not detectable		
	Clone		32.150	**<0.001**		1.808	0.613						

*To account for the variance heterogeneity of the residuals the varIdent variance structure was used. Under “Variance” it is specified whether the variance is controlled for each aphid clone-plant species combination (Treat), for each aphid race (Race) or for each time point (Time). Significant *P*-values are given in bold*.

In *P. sativum*, the SA levels changed less over time. At most time points, SA levels in plants with the native PR clone were equivalent to levels in uninfested control plants. SA levels in plants with non-native aphid clones did not follow a consistent pattern. They were higher (TR at all time points except 72 h, MR at 24 h), lower (MR at 72 h) or similar (MR at 48 h and 96 h, TR at 72 h) than those in uninfested control plants (**Figure 2C**). In contrast, the levels of SA in the universal host *V. faba* did not change very much in the first 72 h after aphid infestation for all aphid clones. However, 96 h after aphid infestation SA levels were significantly higher in aphid infested plants than in uninfested control plants. Whereas the PR clone elicited only a minimal increase, the TR and in particular the MR clone triggered a much higher increase (**Figure 2D**).

In uninfested control plants, JA-Ile levels behaved similarly to SA levels, staying constant over time or changing only slightly compared to changes triggered by aphid infestation. The strength of the aphid-triggered changes was aphid clone dependent (**Figures 2E–H**; **Table 4**). In *M. sativa* and *T. pratense* during the first three time points after aphid infestation, the JA-Ile concentration was significantly higher in plants with non-native clones compared to plants infested with the native clone or uninfested control plants. When infested with the native clone JA-Ile levels in *T. pratense* plants were in the same range as those in uninfested control plants, whereas JA-Ile levels in *M. sativa* were mostly significantly higher than the levels in the uninfested control plants. For both plant species the JA-Ile levels of plants infested with the native aphid clone increased after 72 h and reached similar levels as in plants infested with non-native aphids at 96 h after aphid infestation (**Figures 2E,F**).

When the native PR clone fed on *P. sativum* plants, the JA-Ile levels steadily increased starting from levels comparable with those in uninfested control plants, and ending with levels being significantly higher than in control plants, but lower than in plants infested with non-native aphid clones (MR, TR). Levels in plants infested with non-native aphid clones fluctuated over time, being as low as in control plants (TR at 24 h, MR at 72 h) or significantly higher than in control plants (TR at 48, 72, and 96 h, MR at 24, 48, and 96 h) (**Figure 2G**). In *V. faba* plants, JA-Ile levels increased in all aphid-infested plants from 24 to 48 h being always higher than levels in the control plants. Afterward JA-Ile levels triggered by aphids fluctuated in a clone specific manner over time. At 96 h after aphid infestation, JA-Ile levels in aphid-infested plants were lower (PR- and TR-infested plants), or higher (MR-infested plants) than in uninfested control plants (**Figure 2H**).

Abscisic acid levels fluctuated over time in all four plant species (**Figures 2I–L**; **Table 4**), and fluctuated depending on the aphid clone in all plant species but *V. faba*. There were no differences between native and non-native clones. ABA levels in aphid-infested plants were generally either reduced or were similar to levels in uninfested control plants (**Figures 2I–L**). Only in *M. sativa* 24 h after aphid infestation, ABA levels in aphid-infested plants were higher than in uninfested control plants (**Figure 2I**).

### Clones from Native Host Races Induced Lower Levels of JA-Pathway Metabolites Than Non-native Races

To obtain information about the effect of pea aphid infestation on the formation and further metabolism of the active jasmonate, the JA-Ile conjugate, we measured the levels of its precursors the 12-oxo phytodienoic acid (OPDA), and JA, as well as its metabolites, the 12-hydroxyjasmonic acid-isoleucine conjugate (12-OH-JA-Ile), the 12-carboxyjasmonic acid-isoleucine conjugate (12-COOH-JA-Ile), and two hydroxylated forms of unconjugated JA (OH-JA1 and OH-JA2).

**FIGURE 3:**
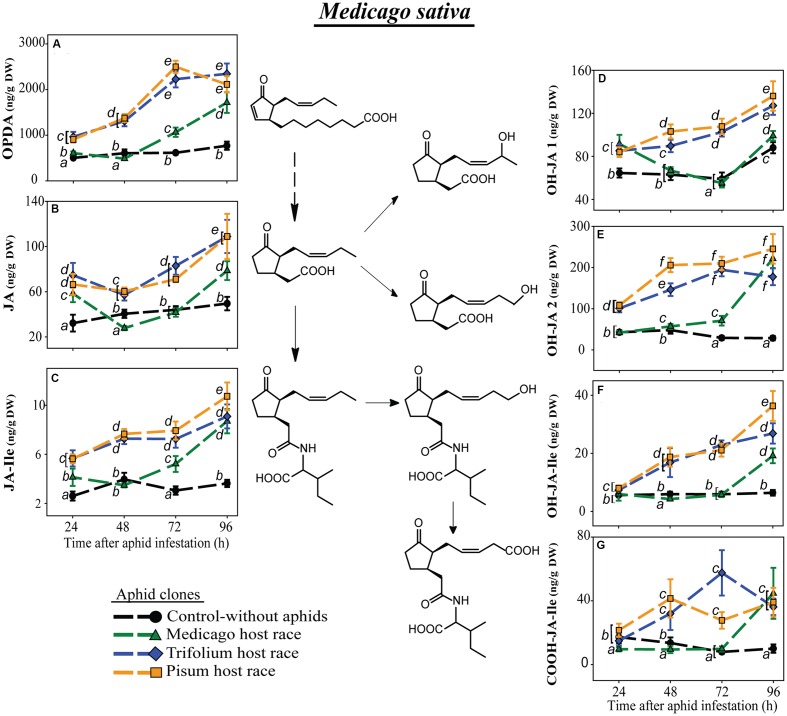
**Level of JA-pathway metabolites in *M. sativa* plants after infestation with pea aphid clones of different host races**. Symbols represent means ± SE. Statistical values are presented in **Table 4**. JA-pathway metabolites are 12-oxo phytodienoic acid (OPDA) **(A)**, jasmonic acid (JA) **(B)**, JA-isoleucine conjugate (JA-Ile) **(C)**, two hydroxyjasmonic acid isomers OH-JA1 **(D)** and OH-JA2 **(E)**, 12-hydroxyjasmonic acid-isoleucine conjugate (OH-JA-Ile) **(F)**, and 12-carboxyjasmonic acid-isoleucine conjugate (COOH-JA-Ile) **(G)**. Different letters indicate significant differences between treatments (*P* ≤ 0.05).

In *M. sativa*, all measured JA-Ile precursors and further metabolites generally had significantly lower levels after infestation with the native clone MR than after the non-native clones TR and PR (**Figure 3**; **Table 4**). This pattern was especially visible for the JA-Ile precursors, OPDA and JA (**Figures 3A,B**). The levels of the hydroxylated and carboxylated forms of JA and JA-Ile were mostly lowest in plants infested with the native MR clone, similar to the levels in uninfested control plants, but increased after 72 h reaching sometimes levels comparable to the ones in plants infested with non-native aphids 96 h after aphid infestation (**Figures 3E–G**).

**FIGURE 4:**
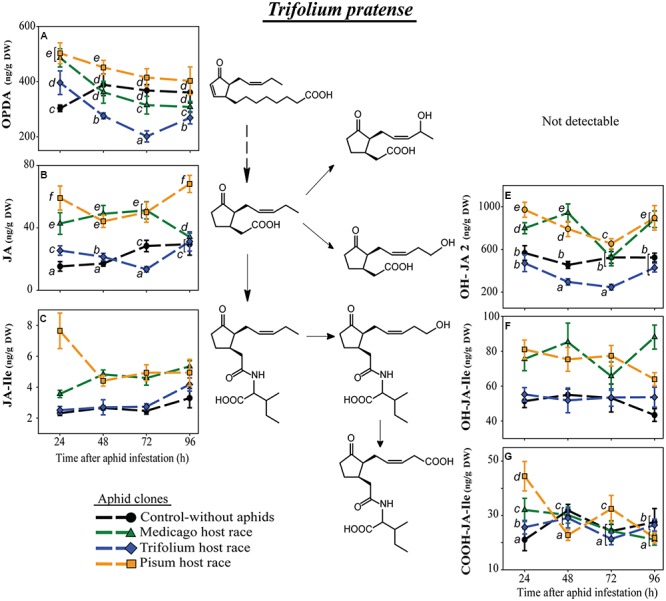
**Level of JA-pathway metabolites in *T. pratense* plants after infestation with pea aphid clones of different host races**. Symbols represent means ± SE. Statistical values are presented in **Table 4**. JA-pathway metabolites are 12-oxo phytodienoic acid (OPDA) **(A)**, jasmonic acid (JA) **(B)**, JA-isoleucine conjugate (JA-Ile) **(C)**, hydroxyjasmonic acid isomer (OH-JA2) **(E)**, 12-hydroxyjasmonic acid-isoleucine conjugate (OH-JA-Ile) **(F)**, and 12-carboxyjasmonic acid-isoleucine conjugate (COOH-JA-Ile) **(G)**. In cases where a significant influence of the aphid clone on the phytohormone level was dependent on the time after aphid infestation (interaction), significant differences (*P* ≤ 0.05) between aphid clones at different time points are indicated by different letters.

Equivalently in *T. pratense*, levels of the precursors of JA-Ile, OPDA, and JA were always significantly lower after infestation with the native TR clone than the non-native clones MR and PR (**Figures 4A,B**). At 24 h after aphid infestation, plants harboring the native aphid clone TR had OPDA levels even below the concentration in uninfested control plants (**Figure 4A**; Supplementary Table S2). This strong downregulation was also visible for OH-JA2 (**Figure 4E**), whereas the other metabolite of JA, OH-JA1, was not detectable in *T. pratense*. Also the levels of the hydroxylated derivatives of JA-Ile were higher in plants infested with the non-native aphid clones (**Figures 4E,F**). Levels of the carboxylated JA-Ile derivative fluctuated without evidence of a specific pattern. Of all the aphid-infested plants those infested with the native aphid clone TR showed levels most similar to the levels in uninfested control plants (**Figure 4G**).

**FIGURE 5:**
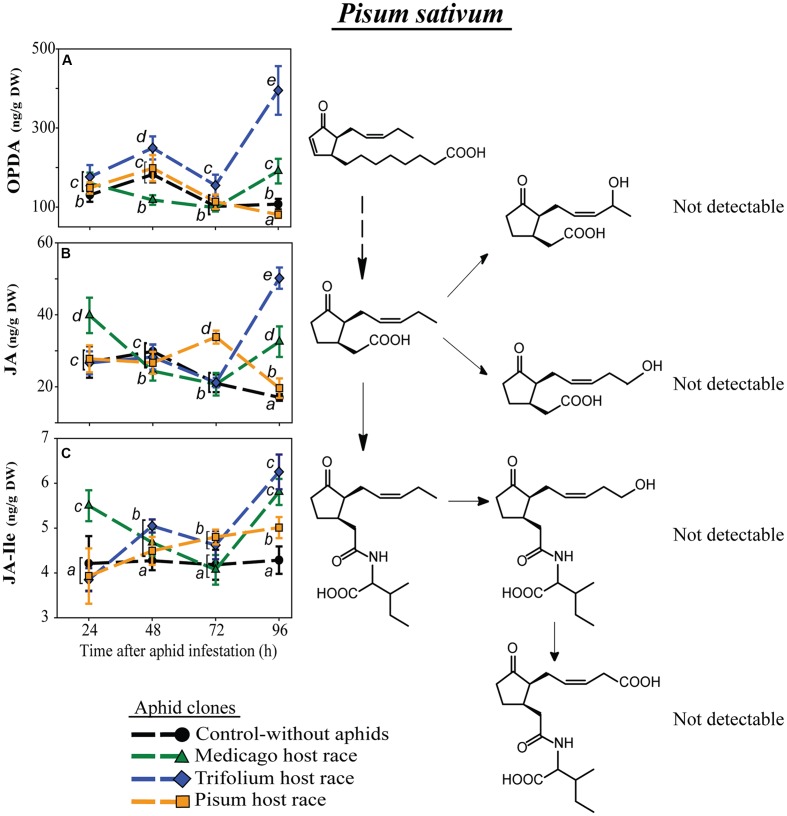
**Level of JA-pathway metabolites in *P. sativum* plants after infestation with pea aphid clones of different host races**. Symbols represent means ± SE. Statistical values are presented in **Table 4**. JA-pathway metabolites are 12-oxo phytodienoic acid (OPDA) **(A)**, jasmonic acid (JA) **(B)**, and JA-isoleucine conjugate (JA-Ile) **(C)**. Different letters indicate significant differences between treatments (*P* ≤ 0.05).

In contrast to the other plant species, *P. sativum* did not possess detectable levels of the metabolized forms of JA or JA-Ile (**Figure 5**). Levels of both JA-Ile precursors, OPDA and JA, changed over time in a clone-specific way (**Figures 5A,B**; **Table 4**) with levels in plants infested with the native PR clone usually being most similar to levels in uninfested control plants.

**FIGURE 6:**
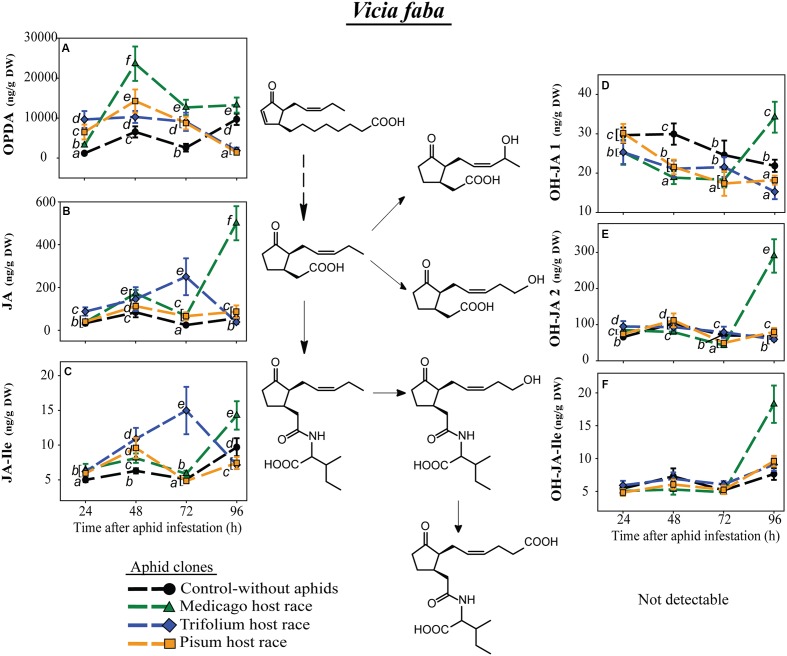
**Level of JA-pathway metabolites in *V. faba* plants after infestation with pea aphid clones of different host races**. Symbols represent means ± SE. Statistical values are presented in **Table 4**. JA-pathway metabolites are 12-oxo phytodienoic acid (OPDA) **(A)**, jasmonic acid (JA) **(B)**, JA-isoleucine conjugate (JA-Ile) **(C)**, two hydroxyjasmonic acid isomers OH-JA1 **(D)** and OH-JA2 **(E)**, and 12-hydroxyjasmonic acid-isoleucine conjugate (OH-JA-Ile) **(F)**. In cases where a significant influence of the aphid clone on the phytohormone level was dependent on the time after aphid infestation (interaction), significant differences (*P* ≤ 0.05) between aphid clones at different time points are indicated by different letters.

In the universal host, *V. faba*, levels of the JA-Ile precursors, OPDA and JA, did change over time but in an aphid clone-specific way. At most time points both precursor levels were higher in plants infested by each of the aphid clones than in uninfested control plants. This difference was much more pronounced for OPDA than for JA (**Figures 6A,B**). However, 96 h after aphid infestation OPDA levels were significantly lower in plants infested with the PR and TR clones than in uninfested control plants, and JA levels were similar to (for PR) or lower than (for TR) in uninfested control plants (**Figures 6A,B**). In contrast, the MR clone caused very high JA levels 96 h after aphid infestation (**Figure 6B**), and this increase carried over to the other JA metabolites detected in MR-infested *V. faba*, JA-Ile, OH-JA1, OH-JA2, and 12-OH-JA-Ile (**Figures 6C–F**). The carboxylated form of JA-Ile, 12-COOH-JA-Ile, could not be detected in *V. faba*. For other aphid clones, levels of JA and JA-Ile metabolites were either decreased by aphid infestation (OH-JA1, **Figure 6D**) or were similar to those in uninfested control plants (OH-JA2, **Figure 6E**, and 12-OH-JA-Ile, **Figure 6F**). There were only a few significant changes in JA and JA-Ile metabolites in the control uninfested plants (e.g., **Figures 6C,D**). The ones that occurred may be ascribed to developmental changes or attempts to mimic the experimental manipulations performed on the infested plants (enclosure in an air-permeable cellophane bag to prevent aphid escape, leaf brushing to remove aphids before sampling) on the controls as well.

## Discussion

### Infestation with Native Pea Aphid Host Races Leads to Lower Jasmonate and Salicylate Signaling

When legume plants were infested with clones of different pea aphid host races, several distinct patterns of phytohormone response were observed depending on the legume species, the pea aphid clone, the compatibility between plant and aphid, and the duration of the aphid infestation. In *T. pratense* and *M. sativa*, the concentration of the active form of the JA, JA-Ile, corresponded well with the aphid performance. Non-native aphids elicited a strong JA-Ile response whereas infestation with native aphid clones led to a much weaker induction (MR on *M. sativa*) or even to a near total absence of JA-Ile induction (TR on *T. pratense*). This weaker induction could be due to a lack of recognition of the aphid by the plant or an active suppression, which seems more likely since the JA pathway is usually activated upon wounding. When aphids initially penetrate plant tissue they regularly pierce and salivate into cells before arriving at the phloem and attempting to feed. Since aphids spend more time in this penetration phase on native than on non-native host plants (Schwarzkopf et al., 2013), they likely also pierce more cells and cause more tissue damage on native hosts. More damage should result in a stronger JA response to native aphid clones than to non-native clones, but this was not the case. Thus aphids on their native host were either able to hide the damage they caused from plant recognition systems or to actively suppress the plant-defense response. The defense suppression hypothesis is also supported from the finding that previous pea aphid infestation resulted in an increased performance of conspecific offspring (Takemoto et al., 2013). Similar effects are known from other aphid species like the soybean aphid *Aphis glycines* (Varenhorst et al., 2015). This conclusion also suggests that a strong up-regulation of JA-defense signaling is responsible for the low performance of non-adapted aphid clones.

The efficacy of JA-defense signaling has been shown in several other plant-aphid interactions. For example, in *A. thaliana* Ellis et al. (2002) recognized an enhanced resistance against *M. persicae* after the activation of the JA pathway. Genetic data also support the idea that the JA-defense pathway is the important one in plant-aphid interactions. Ten out of 13 tested genes associated with the JA pathway were induced only in *Medicago truncatula* plants resistant to *A. kondoi*, and not in susceptible *M. truncatula*, whereas all tested genes related to the SA pathway were induced independently of the susceptibility of the plant (Gao et al., 2007).

The overall negative relation between aphid performance and JA levels was only partially true for SA. For instance, after 96 h on its native host, *M. sativa*, the MR clone elicited a high SA as well as a high JA response just as high or higher than that elicited by the non-native aphid clones, but in contrast to the non-native clones MR aphids survived and reproduced well on *M. sativa*. SA levels or the expression of SA-related genes have often been reported to be upregulated due to aphid infestation (Moran and Thompson, 2001; De Vos and Jander, 2009; Mai et al., 2014; Zhang et al., 2015; Stewart et al., 2016), and so we cannot yet exclude its importance for the pea aphid. Such an SA upregulation can also be triggered by factors derived from aphid endosymbionts, which might enter the plant via insect saliva. This is known for the GroEL protein of the obligate aphid endosymbiont *Buchnera aphidicola* (Chaudhary et al., 2014), which induced SA-defense marker gene expression. Transgenic *A. thaliana* lines expressing GroEL exhibited a significant but small reduction in aphid fecundity. Thus SA-related defense triggered by endosymbionts led to a fitness cost but was not strong enough to prevent aphid increase.

Regardless of whether the JA- or SA-defense pathway was most effective against non-native aphids in our experiments, our measurements of aphid performance and phytohormone levels suggest that the native aphid clones (clone TR on *T. pratense*, clone MR on *M. sativa*) were able to suppress plant defenses on their native host plants (*T. pratense* and *M. sativa*). This suppression may not have been complete since at 96 h after aphid infestation JA-Ile levels of *M. sativa* infested by the native clone equaled levels in most plants infested by non-native clones. Such an increase might be due to the increased number of aphids on the plant, which is known to influence the level of defense signaling (Mai et al., 2014; Stewart et al., 2016). Nevertheless the native MR clone survived and developed on its native host much better than non-native clones indicating its ability to cope with both the constitutive and any induced defense of the plant (Walling, 2008).

A different pattern of phytohormone response was observed in *P. sativum* after pea aphid infestation. This plant is the native host of the PR clone, but the other aphid clones also showed substantial survival, growth and reproduction on this plant (Figure 1, Schwarzkopf et al., 2013). The intermediate performance of non-native clones on *P. sativum* was also reflected in the SA and JA response of the plant. In contrast to the patterns for *M. sativa* and *T. pratense*, non-native aphids did not trigger a strong, consistent induction of JA-Ile and SA over the whole time course, except at 96 h after infestation when the non-native clones elicited higher JA-Ile levels than the native PR clone. Infestation with the non-native clones also caused stronger fluctuations in JA-Ile and SA profiles over time compared to infestation with the native clone. Such fluctuations were also reported for JA and JA methyl ester in *P. sativum* plants after pea aphid infestation (Mai et al., 2014). In the *A. thaliana* – *Brevicoryne brassicae* system, JA-related gene transcripts also showed fluctuations after infestation (Kusnierczyk et al., 2008). Whether these fluctuations were an expression of the intermediate ability of the aphids to deal with the plant response remains an open question. Aphid performance may be a consequence of their influence on plant-defense signaling pathways or their tolerance of defense toxins, deterrents and phloem-sealing mechanisms.

On the universal host plant *V. faba* both the JA- and the SA-regulated plant defenses seemed to be non-effective since clones of all host races performed very well in comparison to on other host plants. That pea aphids can positively influence *V. faba* for their own benefit was already reported by Takemoto et al. (2013), who observed that *A. pisum* nymphs developed faster when they could feed on *V. faba* plants previously infested by pea aphids. Since pre-infested *V. faba* produced less JA than uninfested control plants, the involvement of JA-related defenses was presumed. The pattern of phytohormone changes in this species was different than that for any other host plant. Basal SA levels were much lower than in all the other measured plant species. The levels were low for all clones until the last time point when they rose significantly with respect to those of uninfested control plants, where they reached levels also found in other plant species. Thus, SA signaling did not lead to effective defense against aphids in *V. faba*. JA-Ile levels generally rose over the whole time course, but curiously JA-Ile levels for the TR and PR clones were low at the last time point, even lower than those in the uninfested control. For these clones, the low JA-Ile levels went along with a high performance on *V. faba* at 96 h.

Abscisic acid, a phytohormone long known to regulate plant growth (Cutler et al., 2010), protect against water stress (Schroeder et al., 2001), control seed dormancy and germination (Karssen et al., 1983), and participate in source-sink communication (Yu et al., 2015), has recently been found to be a major modulator of plant defense as well (Mauch-Mani and Mauch, 2005; Ton et al., 2009; Pieterse et al., 2012). ABA has been reported to interact with the JA- and SA-defense pathways. For instance, upon wounding or herbivory ABA acts synergistically with JA on the MYC branch of the JA pathway leading to an increased resistance to herbivory (Anderson et al., 2004; Yasuda et al., 2008). On the other hand, ABA can suppress SA-dependent defenses (De Torres Zabala et al., 2009; Jiang et al., 2010; Cao et al., 2011). Concerning aphids, there are several reports that infestation induced ABA levels or ABA-regulated gene expression in *Glycine max*, *M. truncatula*, and *A. thaliana* (Studham and Macintosh, 2013; Guo et al., 2015; Sun et al., 2015; Hillwig et al., 2016). In contrast, another study showed that ABA levels in *M. truncatula* were not affected or even reduced by *A. pisum* feeding (Stewart et al., 2016) a pattern we also found in our study, where ABA levels in aphid-infested plants were generally lower or very similar than those in control plants. Since this pattern held regardless of the plant or aphid clone studied, ABA does not seem to modulate defense reactions against pea aphids in legumes. However, ABA could play other roles in plant-aphid interactions. For instance, ABA-driven stomatal closure could be advantageous for aphids under dry conditions since it maintains plant turgor and so facilitates aphid feeding (Guo et al., 2015). However, by causing reductions in photosynthetic activity, ABA-induced closure of stomata could decrease the carbohydrate supply available to aphids. Interestingly, among the plant species studied, ABA levels were quite different, ranging from about 50 ng/g DW in TR infested *P. sativum* plants (72 h after aphid infestation) to more than 400 ng/g DW in PR infested *M. sativa* plants (72 h after aphid infestation). Also basal levels of ABA varied a lot between plants which suggest that changes in phytohormone levels between treatments are more important than absolute phytohormone levels.

### Native Pea Aphid Host Races May Block Specific Steps in Jasmonate Signaling or Biosynthesis

To explore the mechanism by which native aphid clones might suppress the increase of JA-Ile, we investigated the levels of JA-Ile precursors and catabolites after infestation of clones of the various host races. Lower JA-Ile levels might result from lower levels of the precursors OPDA and JA, or to increased metabolism of JA-Ile to hydroxylated and carboxylated derivatives (OH-JA-Ile, OH-JA1, OH-JA2, and COOH-JA-Ile), which could inactivate JA signaling (Miersch et al., 2008; Koo and Howe, 2012; Koo et al., 2014).

The levels of OPDA, the first metabolite in the JA pathway that we measured, were different in *M. sativa* and *T. pratense* plants depending on the infesting aphid clone. In plants infested with non-native aphid clones, levels of OPDA were higher than in plants infested with the native clone, consistent with the trends in JA-Ile concentration. In the universal host plant *V. faba* OPDA levels were generally enhanced over the 72 h following aphid infestation. However, at 96 h after infestation, the TR and PR clones suppressed OPDA formation below the levels for uninfested control plants, suggesting that aphids influence the JA pathway prior to the formation of OPDA. The fatty acid substrate of the JA pathway is α-linolenic acid (18:3), which is produced from galactolipids of chloroplast membranes (Wasternack and Hause, 2013). Recently Kanobe et al. (2015) detected less α-linolenic acid in soybean plants (*G. max*) infested with the soybean aphid (*Aphis glycine*) than in uninfested control plants or plants infested with other soybean antagonists, the soybean cyst nematode (*Heterodera glycines*) and the brown stem rot (*Cadophora gregata*). This suggests that certain pea aphid clones might suppress one of the steps in JA signaling or biosynthesis prior to the formation of α-linolenic acid. Or, the site of suppression could follow galactolipid hydrolysis. α-Linolenic acid is converted to OPDA in three steps by the sequential action of lipoxygenase (LOX), allene oxide cyclase (AOC) and allene oxide synthase (AOS) (Wasternack and Hause, 2013). The activity of LOX increased upon aphid infestation (Mai et al., 2014), while the genes encoding LOX and AOS were upregulated more strongly in wheat infested by an incompatible biotype of the Russian wheat aphid (*Diuraphis noxia*) than in wheat infested by a compatible biotype (Liu et al., 2011). Thus compatible (native) pea aphid biotypes might suppress OPDA levels by downregulating the activities of LOX or AOS.

Aphids might also reduce JA-Ile levels by accelerating catabolism to hydroxylated and carboxylated derivatives. These metabolites might additionally contribute to a partial switch-off of JA signaling (Miersch et al., 2008). In our experiments, the abundance of JA and JA-Ile metabolites was generally correlated with that of JA and JA-Ile making it unlikely that native host races owe their suppression of JA signaling to upregulation of jasmonate catabolism. In addition, jasmonate metabolite levels were often higher in plants infested with non-adapted than adapted clones. Interestingly, among the plant species studied, there was large variation in the levels of the jasmonate metabolites. For instance, *P. sativum* did not contain JA or JA-Ile metabolites in detectable amounts, while they were highest, especially OH-JA2, in *T. pratense*. *P. sativum* might use other metabolic conversions to fine tune the JA pathway, like the methylation of JA and JA-Ile resulting in methyl-JA and methyl-JA-Ile, or glycosylation leading to JA-glucoside and JA-Ile-glucoside (Gfeller et al., 2010; Koo and Howe, 2012) – compounds which were not measured in this study. Taking the species together, when the hydroxylated and carboxylated metabolites were present, their levels were of the same magnitude as JA, whereas JA-Ile was present in levels an order of magnitude lower while OPDA was present at levels 1–2 orders of magnitude higher. However, this inter-plant variation in JA metabolites may only partially represent the true differences among the species. Other JA and JA-Ile metabolites, such as methylated or glucosylated forms of JA and JA-Ile, and other JA-amino acid conjugates are known (Gfeller et al., 2010; Koo and Howe, 2012) and might occur in legumes as well.

## Conclusion

While plants deploy many different modes of defense against aphids (Edwards and Singh, 2006; Züst and Agrawal, 2016), aphids often feed readily on their host plants. Yet our knowledge of the mechanisms by which aphids circumvent plant defenses is still quite limited. In the pea aphid complex, we have now shown that the ability of host races to feed on their native host plants may lie in their ability to manipulate defense signaling pathways either by avoiding recognition or by suppressing JA and SA signaling much more effectively on their native hosts than on non-native plants. Strikingly, this reduced JA and SA signaling triggered by native races occurred even though plant damage on native hosts was much higher due to a greater aphid population density resulting from higher growth, survival and reproduction rates. Since lower levels of the active JA-Ile conjugate were correlated with lower levels of the other JA-pathway metabolites measured (OPDA, JA, various hydroxylated and carboxylated derivatives), native host races likely block jasmonate formation upstream of OPDA. Plant ABA concentration did not change according to the native or non-native status of the infesting aphid clone indicating that ABA does not make a large contribution to the differential ability of pea aphid host races to colonize a plant.

The low levels of JA and SA in plants infested with native pea aphid host races were combined with significantly better performance. Hence native races may be able to reduce plant defenses, such as toxins, deterrents, and phloem-sealing mechanisms. Further work is necessary to identify these defense mechanisms. Additional research is also needed to understand the cause of reduced defense signals. Previous aphid work has often focused on the salivary effector proteins that are injected into host plants and the way these modulate plant processes to facilitate feeding (Hogenhout and Bos, 2011; Pitino and Hogenhout, 2013). Since all pea aphid clones, both native and non-native, are able to begin penetrating the plant (Schwarzkopf et al., 2013), but only some are able to feed and perform well, the type and quantity of these effectors may be critical in modulating plant-defense signaling and mediating aphid success. Future work on the nature of these effectors and the differences among pea aphid host races may help identify the basis for differential defense signaling.

## Author Contributions

CS-A and GK conceived and designed the experiments. CS-A performed the experiments. CS-A and MR executed the phytohormone analyses. CS-A and GK analyzed data. CS-A, GK, and JG interpreted the results and wrote the manuscript. All authors critically revised and consented to the final version of the manuscript.

## Conflict of Interest Statement

The authors declare that the research was conducted in the absence of any commercial or financial relationships that could be construed as a potential conflict of interest.

## References

[B1] Abdellatef E. Will T. Koch A. Imani J. Vilcinskas A. Kogel K.-H. (2015). Silencing the expression of the salivary sheath protein causes transgenerational feeding suppression in the aphid *Sitobion avenae*. *Plant Biotechnol. J.* 13 849–857. 10.1111/pbi.12322 25586210

[B2] Anderson J. P. Badruzsaufari E. Schenk P. M. Manners J. M. Desmond O. J. Ehlert C. (2004). Antagonistic interaction between abscisic acid and jasmonate-ethylene signaling pathways modulates defense gene expression and disease resistance in *Arabidopsis*. *Plant Cell* 16 3460–3479. 10.1105/tpc.104.025833 15548743 PMC535886

[B3] Beckers G. J. M. Spoel S. H. (2006). Fine-tuning plant defence signalling: salicylate versus jasmonate. *Plant Biol.* 8 1–10. 10.1055/s-2005-872705 16435264

[B4] Blackman R. L. Eastop V. F. (2000). *Aphids on the World’s Crops.* Chichester: Wiley.

[B5] Bos J. I. B. Prince D. Pitino M. Maffei M. E. Win J. Hogenhout S. A. (2010). A functional genomics approach identifies candidate effectors from the aphid species *Myzus persicae* (Green peach aphid). *PLoS Genet.* 6:e1001216 10.1371/journal.pgen.1001216 PMC2987835 21124944

[B6] Brooks D. M. Bender C. L. Kunkel B. N. (2005). The *Pseudomonas syringae* phytotoxin coronatine promotes virulence by overcoming salicylic acid-dependent defences in *Arabidopsis thaliana*. *Mol. Plant Pathol.* 6 629–639. 10.1111/j.1364-3703.2005.00311.x 20565685

[B7] Caarls L. Pieterse C. M. J. Van Wees S. C. M. (2015). How salicylic acid takes transcriptional control over jasmonic acid signaling. *Front. Plant Sci.* 6:170 10.3389/fpls.2015.00170 PMC4373269 25859250

[B8] Cao F. Y. Yoshioka K. Desveaux D. (2011). The roles of ABA in plant–pathogen interactions. *J. Plant Res.* 124 489–499. 10.1007/s10265-011-0409-y 21380629

[B9] Carolan J. C. Caragea D. Reardon K. T. Mutti N. S. Dittmer N. Pappan K. (2011). Predicted effector molecules in the salivary secretome of the pea aphid (*Acyrthosiphon pisum*): a dual transcriptomic/proteomic approach. *J. Proteome Res.* 10 1505–1518. 10.1021/pr100881q 21226539

[B10] Carolan J. C. Fitzroy C. I. J. Ashton P. D. Douglas A. E. Wilkinson T. L. (2009). The secreted salivary proteome of the pea aphid *Acyrthosiphon pisum* characterised by mass spectrometry. *Proteomics* 9 2457–2467. 10.1002/pmic.200800692 19402045

[B11] Chaudhary R. Atamian H. S. Shen Z. Briggs S. P. Kaloshian I. (2014). GroEL from the endosymbiont *Buchnera aphidicola* betrays the aphid by triggering plant defense. *Proc. Natl. Acad. Sci. U.S.A.* 111 8919–8924. 10.1073/pnas.1407687111 24927572 PMC4066539

[B12] Chen Z. X. Silva H. Klessig D. F. (1993). Active oxygen species in the induction of plant systemic acquired resistance by salicylic acid. *Science* 262 1883–1886. 10.1126/science.8266079 8266079

[B13] Crawley M. (2013). *The R Book.* Hoboken, NJ: John Wiley and Sons, Ltd.

[B14] Cutler S. R. Rodriguez P. L. Finkelstein R. R. Abrams S. R. (2010). Abscisic acid: emergence of a core signaling network. *Annu. Rev. Plant Biol.* 61 651–679. 10.1146/annurev-arplant-042809-112122 20192755

[B15] De Torres Zabala M. Bennett M. H. Truman W. H. Grant M. R. (2009). Antagonism between salicylic and abscisic acid reflects early host–pathogen conflict and moulds plant defence responses. *Plant J.* 59 375–386. 10.1111/j.1365-313X.2009.03875.x 19392690

[B16] De Vos M. Jander G. (2009). *Myzus persicae* (green peach aphid) salivary components induce defence responses in *Arabidopsis thaliana*. *Plant Cell Environ.* 32 1548–1560. 10.1111/j.1365-3040.2009.02019.x 19558622

[B17] De Vos M. Van Oosten V. R. Van Poecke R. M. P. Van Pelt J. A. Pozo M. J. Mueller M. J. (2005). Signal signature and transcriptome changes of *Arabidopsis* during pathogen and insect attack. *Mol. Plant Microbe Interact.* 18 923–937. 10.1094/MPMI-18-0923 16167763

[B18] Denance N. Sanchez-Vallet A. Goffner D. Molina A. (2013). Disease resistance or growth: the role of plant hormones in balancing immune responses and fitness costs. *Front. Plant Sci.* 4:155 10.3389/fpls.2013.00155 PMC3662895 23745126

[B19] Diehl S. R. Bush G. L. (1984). An evolutionary and applied perspective of insect biotypes. *Annu. Rev. Entomol.* 29 471–504. 10.1146/annurev.en.29.010184.002351

[B20] Dres M. Mallet J. (2002). Host races in plant-feeding insects and their importance in sympatric speciation. *Philos. Trans. R. Soc. B Biol. Sci.* 357 471–492. 10.1098/rstb.2002.1059 PMC1692958 12028786

[B21] Edwards O. Singh K. B. (2006). Resistance to insect pests: what do legumes have to offer? *Euphytica* 147 273–285. 10.1007/s10681-006-3608-1

[B22] Ellis C. Karafyllidis I. Turner J. G. (2002). Constitutive activation of jasmonate signaling in an *Arabidopsis* mutant correlates with enhanced resistance to *Erysiphe cichoracearum*, *Pseudomonas syringae*, and *Myzus persicae*. *Mol. Plant Microbe Interact.* 15 1025–1030. 10.1094/MPMI.2002.15.10.1025 12437300

[B23] Elzinga D. A. De Vos M. Jander G. (2014). Suppression of plant defenses by a *Myzus persicae* (Green peach aphid) salivary effector protein. *Mol. Plant Microbe Interact.* 27 747–756. 10.1094/MPMI-01-14-0018-R 24654979 PMC4170801

[B24] Ferrari J. Godfray H. C. J. Faulconbridge A. S. Prior K. Via S. (2006). Population differentiation and genetic variation in host choice among pea aphids from eight host plant genera. *Evolution* 60 1574–1584. 10.1111/j.0014-3820.2006.tb00502.x 17017058

[B25] Ferrari J. Via S. Godfray H. C. J. (2008). Population differentiation and genetic variation inperformance on eight hosts in the pea aphid complex. *Evolution* 62 2508–2524. 10.1111/j.1558-5646.2008.00468.x 18647340

[B26] Finkelstein R. (2013). Abscisic acid synthesis and response. *Arabid. Book* 11:e0166 10.1199/tab.0166 PMC3833200 24273463

[B27] Furch A. C. U. Van Bel A. J. E. Will T. (2015). Aphid salivary proteases are capable of degrading sieve-tube proteins. *J. Exp. Bot.* 66 533–539. 10.1093/jxb/eru487 25540441

[B28] Gao L.-L. Anderson J. P. Klingler J. P. Nair R. M. Edwards O. R. Singh K. B. (2007). Involvement of the octadecanoid pathway in bluegreen aphid resistance in *Medicago truncatula*. *Mol. Plant Microbe Interact.* 20 82–93. 10.1094/MPMI-20-0082 17249425

[B29] Gao L.-L. Klingler J. P. Anderson J. P. Edwards O. R. Singh K. B. (2008). Characterization of pea aphid resistance in *Medicago truncatula*. *Plant Physiol.* 146 996–1009. 10.1104/pp.107.111971 18184733 PMC2259086

[B30] Gfeller A. Dubugnon L. Liechti R. Farmer E. E. (2010). Jasmonate biochemical pathway. *Sci. Signal.* 3:cm3 10.1126/scisignal.3109cm3 20159849

[B31] Gimenez-Ibanez S. Solano R. (2013). Nuclear jasmonate and salicylate signaling and crosstalk in defense against pathogens. *Front. Plant Sci.* 4:72 10.3389/fpls.2013.00072 PMC3617366 23577014

[B32] Giordanengo P. Brunissen L. Rusterucci C. Vincent C. Van Bel A. Dinant S. (2010). Compatible plant-aphid interactions: how aphids manipulate plant responses. *C. R. Biol.* 333 516–523. 10.1016/j.crvi.2010.03.007 20541163

[B33] Goggin F. L. (2007). Plant-aphid interactions: molecular and ecological perspectives. *Curr. Opin. Plant Biol.* 10 399–408. 10.1016/j.pbi.2007.06.004 17652010

[B34] Guo H. Sun Y. Peng X. Wang Q. Harris M. Ge F. (2015). Up-regulation of abscisic acid signaling pathway facilitates aphid xylem absorption and osmoregulation under drought stress. *J. Exp. Bot.* 67 681–693. 10.1093/jxb/erv481 26546578 PMC4737068

[B35] Hawthorne D. J. Via S. (2001). Genetic linkage of ecological specialization and reproductive isolation in pea aphids. *Nature* 412 904–907. 10.1038/35091062 11528477

[B36] Herrera-Vasquez A. Salinas P. Holuigue L. (2015). Salicylic acid and reactive oxygen species interplay in the transcriptional control of defense genes expression. *Front. Plant Sci.* 6:171 10.3389/fpls.2015.00171 PMC4365548 25852720

[B37] Hillwig M. S. Chiozza M. Casteel C. L. Lau S. T. Hohenstein J. Hernández E. (2016). Abscisic acid deficiency increases defence responses against *Myzus persicae* in Arabidopsis. *Mol. Plant Pathol.* 17 225–235. 10.1111/mpp.12274 25943308 PMC6638517

[B38] Hogenhout S. A. Bos J. I. B. (2011). Effector proteins that modulate plant-insect interactions. *Curr. Opin. Plant Biol* 14 422–428. 10.1016/j.pbi.2011.05.003 21684190

[B39] Howe G. A. (2004). Jasmonates as signals in the wound response. *J. Plant Growth Regul.* 23 223–237. 10.1007/s00344-004-0030-6

[B40] Howe G. A. Jander G. (2008). Plant immunity to insect herbivores. *Annu. Rev. Plant Biol.* 59 41–66. 10.1146/annurev.arplant.59.032607.092825 18031220

[B41] Jaouannet M. Rodriguez P. A. Thorpe P. Lenoir C. J. G. Macleod R. Escudero-Martinez C. (2014). Plant immunity in plant-aphid interactions. *Front. Plant Sci.* 5:663 10.3389/fpls.2014.00663 PMC4249712 25520727

[B42] Jaquiery J. Stoeckel S. Nouhaud P. Mieuzet L. Maheo F. Legeai F. (2012). Genome scans reveal candidate regions involved in the adaptation to host plant in the pea aphid complex. *Mol. Ecol.* 21 5251–5264. 10.1111/mec.12048 23017212

[B43] Jiang C.-J. Shimono M. Sugano S. Kojima M. Yazawa K. Yoshida R. (2010). Abscisic acid interacts antagonistically with salicylic acid signaling pathway in rice–*Magnaporthe grisea* interaction. *Mol. Plant Microbe Interact.* 23 791–798. 10.1094/MPMI-23-6-0791 20459318

[B44] Jimenez-Aleman G. H. Machado R. A. R. Gorls H. Baldwin I. T. Boland W. (2015). Synthesis, structural characterization and biological activity of two diastereomeric JA-Ile macrolactones. *Organ. Biomol. Chem.* 13 5885–5893. 10.1039/C5OB00362H 25806705

[B45] Kaloshian I. Walling L. L. (2016). Hemipteran and dipteran pests: effectors and plant host immune regulators. *J. Integr. Plant Biol.* 58 350–361. 10.1111/jipb.12438 26467026

[B46] Kamphuis L. G. Zulak K. Gao L.-L. Anderson J. Singh K. B. (2013). Plant-aphid interactions with a focus on legumes. *Funct. Plant Biol.* 40 1271–1284. 10.1071/FP13090 32481194

[B47] Kanobe C. Mccarville M. T. O’Neal M. E. Tylka G. L. Macintosh G. C. (2015). Soybean aphid infestation induces changes in fatty acid metabolism in soybean. *PLoS ONE* 10:e0145660 10.1371/journal.pone.0145660 PMC4684210 26684003

[B48] Karssen C. M. Brinkhorst-Van Der Swan D. L. C. Breekland A. E. Koornneef M. (1983). Induction of dormancy during seed development by endogenous abscisic acid: studies on abscisic acid deficient genotypes of *Arabidopsis thaliana* (L.) Heynh. *Planta* 157 158–165. 10.1007/BF00393650 24264070

[B49] Koo A. J. Howe G. A. (2012). Catabolism and deactivation of the lipid-derived hormone jasmonoyl-isoleucine. *Front. Plant Sci.* 3:19 10.3389/fpls.2012.00019 PMC3355578 22639640

[B50] Koo A. J. Thireault C. Zemelis S. Poudel A. N. Zhang T. Kitaoka N. (2014). Endoplasmic reticulum-associated inactivation of the hormone jasmonoyl-L-isoleucine by multiple members of the cytochrome P450 94 family in Arabidopsis. *J. Biol. Chem.* 289 29728–29738. 10.1074/jbc.M114.603084 25210037 PMC4207986

[B51] Koornneef A. Leon-Reyes A. Ritsema T. Verhage A. Den Otter F. C. Van Loon L. C. (2008). Kinetics of salicylate-mediated suppression of jasmonate signaling reveal a role for redox modulation. *Plant Physiol.* 147 1358–1368. 10.1104/pp.108.121392 18539774 PMC2442557

[B52] Koornneef A. Pieterse C. M. J. (2008). Cross talk in defense signaling. *Plant Physiol.* 146 839–844. 10.1104/pp.107.112029 18316638 PMC2259093

[B53] Kramell R. Schmidt J. Schneider G. Sembdner G. Schreiber K. (1988). Synthesis of N-(jasmonyl)amino acid conjugates. *Tetrahedron* 44 5791–5807. 10.1016/S0040-4020(01)81437-X

[B54] Kusnierczyk A. Winge P. Jorstad T. S. Troczynska J. Rossiter J. T. Bones A. M. (2008). Towards global understanding of plant defence against aphids - timing and dynamics of early *Arabidopsis* defence responses to cabbage aphid (*Brevicoryne brassicae*) attack. *Plant Cell Environ.* 31 1097–1115. 10.1111/j.1365-3040.2008.01823.x 18433442

[B55] Leon-Reyes A. Does D. Lange E. Delker C. Wasternack C. Wees S. M. (2010). Salicylate-mediated suppression of jasmonate-responsive gene expression in *Arabidopsis* is targeted downstream of the jasmonate biosynthesis pathway. *Planta* 232 1423–1432. 10.1007/s00425-010-1265-z 20839007 PMC2957573

[B56] Li Q. Xie Q. G. Smith-Becker J. Navarre D. A. Kaloshian I. (2006). Mi-1-mediated aphid resistance involves salicylic acid and mitogen-activated protein kinase signaling cascades. *Mol. Plant Microbe Interact.* 19 655–664. 10.1094/MPMI-19-0655 16776299

[B57] Liu X. Meng J. Starkey S. Smith C. M. (2011). Wheat gene expression is differentially affected by a virulent russian wheat aphid biotype. *J. Chem. Ecol.* 37 472–482. 10.1007/s10886-011-9949-9 21499720

[B58] Mai V. C. Bednarski W. Borowiak-Sobkowiak B. Wilkaniec B. Samardakiewicz S. Morkunas I. (2013). Oxidative stress in pea seedling leaves in response to *Acyrthosiphon pisum* infestation. *Phytochemistry* 93 49–62. 10.1016/j.phytochem.2013.02.011 23566717

[B59] Mai V. C. Drzewiecka K. Jeleń H. Narożna D. Rucińska-Sobkowiak R. Kęsy J. (2014). Differential induction of *Pisum sativum* defense signaling molecules in response to pea aphid infestation. *Plant Sci.* 22 1–12. 10.1016/j.plantsci.2014.01.011 24656330

[B60] Mauch-Mani B. Mauch F. (2005). The role of abscisic acid in plant–pathogen interactions. *Curr. Opin. Plant Biol.* 8 409–414. 10.1016/j.pbi.2005.05.015 15939661

[B61] Miersch O. Neumerkel J. Dippe M. Stenzel I. Wasternack C. (2008). Hydroxylated jasmonates are commonly occurring metabolites of jasmonic acid and contribute to a partial switch-off in jasmonate signaling. *New Phytol.* 177 114–127. 17995915 10.1111/j.1469-8137.2007.02252.x

[B62] Moran P. J. Thompson G. A. (2001). Molecular responses to aphid feeding in *Arabidopsis* in relation to plant defense pathways. *Plant Physiol.* 125 1074–1085. 10.1104/pp.125.2.1074 11161062 PMC64906

[B63] Morkunas I. Mai V. C. Gabrys B. (2011). Phytohormonal signaling in plant responses to aphid feeding. *Acta Physiol. Plant.* 33 2057–2073. 10.1007/s11738-011-0751-7

[B64] Mur L. A. J. Kenton P. Atzorn R. Miersch O. Wasternack C. (2006). The outcomes of concentration-specific interactions between salicylate and jasmonate signaling include synergy, antagonism, and oxidative stress leading to cell death. *Plant Physiol* 140 249–262. 10.1104/pp.105.072348 16377744 PMC1326048

[B65] Mutti N. S. Louis J. Pappan L. K. Pappan K. Begum K. Chen M.-S. (2008). A protein from the salivary glands of the pea aphid, *Acyrthosiphon pisum*, is essential in feeding on a host plant. *Proc. Natl. Acad. Sci. U.S.A.* 105 9965–9969. 10.1073/pnas.0708958105 18621720 PMC2481341

[B66] Mutti N. S. Park Y. Reese J. C. Reeck G. R. (2006). RNAi knockdown of a salivary transcript leading to lethality in the pea aphid, *Acyrthosiphon pisum. J Insect Sci.* 6 1–7. 10.1673/031.006.3801 PMC2990334 20233093

[B67] Nakamura Y. Mithofer A. Kombrink E. Boland W. Hamamoto S. Uozumi N. (2011). 12-Hydroxyjasmonic acid glucoside is a COI1-JAZ-independent activator of leaf-closing movement in *Samanea saman*. *Plant Physiol.* 155 1226–1236. 10.1104/pp.110.168617 21228101 PMC3046581

[B68] Nomura K. Melotto M. He S.-Y. (2005). Suppression of host defense in compatible plant–*Pseudomonas syringae* interactions. *Curr. Opin. Plant Biol.* 8 361–368. 10.1016/j.pbi.2005.05.005 15936244

[B69] Peccoud J. Maheo F. De La Huerta M. Laurence C. Simon J.-C. (2015). Genetic characterisation of new host-specialised biotypes and novel associations with bacterial symbionts in the pea aphid complex. *Insect Conserv. Divers.* 8 484–492. 10.1111/icad.12131

[B70] Peccoud J. Ollivier A. Plantegenest M. Simon J.-C. (2009a). A continuum of genetic divergence from sympatric host races to species in the pea aphid complex. *Proc. Natl. Acad. Sci. U.S.A.* 106 7495–7500. 10.1073/pnas.0811117106 19380742 PMC2678636

[B71] Peccoud J. Simon J. C. Mclaughlin H. J. Moran N. A. (2009b). Post-Pleistocene radiation of the pea aphid complex revealed by rapidly evolving endosymbionts. *Proc. Natl. Acad. Sci. U.S.A.* 106 16315–16320. 10.1073/pnas.0905129106 19805299 PMC2752580

[B72] Pieterse C. M. J. Leon-Reyes A. Van Der Ent S. Van Wees S. C. M. (2009). Networking by small-molecule hormones in plant immunity. *Nat. Chem. Biol.* 5 308–316. 10.1038/nchembio.164 19377457

[B73] Pieterse C. M. J. Van Der Does D. Zamioudis C. Leon-Reyes A. Van Wees S. C. M. (2012). Hormonal modulation of plant immunity. *Annu. Rev. Cell Dev. Biol.* 28 489–521. 10.1146/annurev-cellbio-092910-154055 22559264

[B74] Pinheiro J. Bates D. Debroy S. Sarkar D. R Core Team (2015). *Nlme: Linear and Nonlinear Mixed Effects Models R package”.* Version 3 1–122. Available at: http://cran.r-project.org/web/packages/nlme/index.html

[B75] Pitino M. Coleman A. D. Maffei M. E. Ridout C. J. Hogenhout S. A. (2011). Silencing of aphid genes by dsRNA feeding from plants. *PLoS ONE* 6:e25709 10.1371/journal.pone.0025709 PMC3187792 21998682

[B76] Pitino M. Hogenhout S. A. (2013). Aphid protein effectors promote aphid colonization in a plant species-specific manner. *Mol. Plant Microbe Interact.* 26 130–139. 10.1094/MPMI-07-12-0172-FI 23035913

[B77] R Development Core Team (2015). *R: A language and Environment for Statistical Computing.* Vienna: R Foundation for Statistical Computing.

[B78] Schenk P. M. Kazan K. Wilson I. Anderson J. P. Richmond T. Somerville S. C. (2000). Coordinated plant defense responses in *Arabidopsis* revealed by microarray analysis. *Proc. Natl. Acad. Sci. U.S.A.* 97 11655–11660. 10.1073/pnas.97.21.11655 11027363 PMC17256

[B79] Schroeder J. I. Kwak J. M. Allen G. J. (2001). Guard cell abscisic acid signalling and engineering drought hardiness in plants. *Nature* 410 327–330. 10.1038/35066500 11268200

[B80] Schwarzkopf A. Rosenberger D. Niebergall M. Gershenzon J. Kunert G. (2013). To feed or not to feed: plant factors located in the epidermis, mesophyll, and sieve elements influence pea aphid’s ability to feed on legume species. *PLoS ONE* 8:e75298 10.1371/journal.pone.0075298 PMC3787088 24098691

[B81] Shabab M. Khan S. A. Vogel H. Heckel D. G. Boland W. (2014). OPDA isomerase GST16 is involved in phytohormone detoxification and insect development. *FEBS J.* 281 2769–2783. 10.1111/febs.12819 24730650

[B82] Simon J.-C. D’Alencon E. Guy E. Jacquin-Joly E. Jaquiery J. Nouhaud P. (2015). Genomics of adaptation to host-plants in herbivorous insects. *Brief. Funct. Genom.* 14 413–423. 10.1093/bfgp/elv015 25846754

[B83] Smith C. M. Boyko E. V. (2007). The molecular bases of plant resistance and defense responses to aphid feeding: current status. *Entomol. Exp. Appl.* 122 1–16. 10.1111/j.1570-7458.2006.00503.x

[B84] Stewart S. A. Hodge S. Bennett M. Mansfield J. W. Powell G. (2016). Aphid induction of phytohormones in *Medicago truncatula* is dependent upon time post-infestation, aphid density and the genotypes of both plant and insect. *Arthropod Plant Interact.* 10 41–53. 10.1007/s11829-015-9406-8

[B85] Stitz M. Gase K. Baldwin I. T. Gaquerel E. (2011). Ectopic expression of AtJMT in *Nicotiana attenuata*: creating a metabolic sink has tissue-specific consequences for the jasmonate metabolic network and silences downstream gene expression. *Plant Physiol.* 157 341–354. 10.1104/pp.111.178582 21753114 PMC3165883

[B86] Studham M. E. Macintosh G. C. (2013). Multiple phytohormone signals control the transcriptional response to soybean aphid infestation in susceptible and resistant soybean plants. *Mol. Plant Microbe Interact.* 26 116–129. 10.1094/MPMI-05-12-0124-FI 22992001

[B87] Sun Y. Guo H. Yuan L. Wei J. Zhang W. Ge F. (2015). Plant stomatal closure improves aphid feeding under elevated CO2. *Global Change Biol.* 10.1111/gcb.12858 [Epub ahead of print]. 25581722

[B88] Takemoto H. Uefune M. Ozawa R. Arimura G.-I. Takabayashi J. (2013). Previous infestation of pea aphids *Acyrthosiphon pisum* on broad bean plants resulted in the increased performance of conspecific nymphs on the plants. *J. Plant Interact.* 8 370–374. 10.1080/17429145.2013.786792

[B89] The International Aphid Genomics Consortium (2010). Genome sequence of the pea aphid *Acyrthosiphon pisum*. *PLoS Biol.* 8:e1000313 10.1371/journal.pbio.1000313 PMC2826372 20186266

[B90] Ton J. Flors V. Mauch-Mani B. (2009). The multifaceted role of ABA in disease resistance. *Trends Plant Sci.* 14 310–317. 10.1016/j.tplants.2009.03.006 19443266

[B91] Van Der Does D. Leon-Reyes A. Koornneef A. Van Verk M. C. Rodenburg N. Pauwels L. (2013). Salicylic acid suppresses jasmonic acid signaling downstream of SCFCOI1-JAZ by targeting GCC promoter motifs via transcription factor ORA59. *Plant Cell* 25 744–761. 10.1105/tpc.112.108548 23435661 PMC3608790

[B92] Vandermoten S. Harmel N. Mazzucchelli G. De Pauw E. Haubruge E. Francis F. (2014). Comparative analyses of salivary proteins from three aphid species. *Insect Mol. Biol.* 23 67–77. 10.1111/imb.12061 24382153

[B93] Varenhorst A. J. Mccarville M. T. O’Neal M. E. (2015). Determining the duration of *Aphis glycines* (Hemiptera: Aphididae) induced susceptibility effect in soybean. *Arthropod. Plant Interact.* 9 457–464. 10.1007/s11829-015-9395-7

[B94] Walling L. L. (2008). Avoiding effective defenses: strategies employed by phloem-feeding insects. *Plant Physiol.* 146 859–866. 10.1104/pp.107.113142 18316641 PMC2259051

[B95] Wang W. Dai H. Zhang Y. Chandrasekar R. Luo L. Hiromasa Y. (2015). Armet is an effector protein mediating aphid-plant interactions. *FASEB J.* 29 2032–2045. 10.1096/fj.14-266023 25678626

[B96] Wasternack C. Hause B. (2013). Jasmonates: biosynthesis, perception, signal transduction and action in plant stress response, growth and development. An update to the 2007 review in Annals of Botany. *Ann. Bot.* 111 1021–1058. 23558912 10.1093/aob/mct067 PMC3662512

[B97] Will T. Furch A. C. U. Zimmermann M. R. (2013). How phloem-feeding insects face the challenge of phloem-located defenses. *Front. Plant Sci.* 4:336 10.3389/fpls.2013.00336 PMC3756233 24009620

[B98] Will T. Tjallingii W. F. Thonnessen A. Van Bel A. J. E. (2007). Molecular sabotage of plant defense by aphid saliva. *Proc. Natl. Acad. Sci. U.S.A.* 104 10536–10541. 10.1073/pnas.0703535104 17553961 PMC1965548

[B99] Wu J. Baldwin I. T. (2010). New insights into plant responses to the attack from insect herbivores. *Annu. Rev. Genet.* 44 1–24. 10.1146/annurev-genet-102209-163500 20649414

[B100] Yasuda M. Ishikawa A. Jikumaru Y. Seki M. Umezawa T. Asami T. (2008). Antagonistic interaction between systemic acquired resistance and the abscisic acid–mediated abiotic stress response in *Arabidopsis*. *Plant Cell* 20 1678–1692. 10.1105/tpc.107.054296 18586869 PMC2483369

[B101] Yu S.-M. Lo S.-F. Ho T.-H. D. (2015). Source-sink communication: regulated by hormone, nutrient, and stress cross-signaling. *Trends Plant Sci.* 20 844–857. 10.1016/j.tplants.2015.10.009 26603980

[B102] Zhang P.-J. Li W.-D. Huang F. Zhang J.-M. Xu F.-C. Lu Y.-B. (2013). Feeding by whiteflies suppresses downstream jasmonic acid signaling by eliciting salicylic acid signaling. *J. Chem. Ecol.* 39 612–619. 10.1007/s10886-013-0283-2 23604702

[B103] Zhang P.-J. Zheng S.-J. Van Loon J. J. A. Boland W. David A. Mumm R. (2009). Whiteflies interfere with indirect plant defense against spider mites in Lima bean. *Proc. Natl. Acad. Sci. U.S.A.* 106 21202–21207. 10.1073/pnas.0907890106 19965373 PMC2795486

[B104] Zhang X. Xue M. Zhao H. (2015). Species-specific effects on salicylic acid content and subsequent Myzus persicae (Sulzer) performance by three phloem-sucking insects infesting *Nicotiana tabacum* L. *Arthropod Plant Interact.* 9 383–391. 10.1007/s11829-015-9385-9

[B105] Züst T. Agrawal A. A. (2016). Mechanisms and evolution of plant resistance to aphids. *Nat. Plants* 2:15206 10.1038/nplants.2015.206 27250753

[B106] Zuur A. Fleno E. N. Walker N. Saveliev A. Smith G. M. (2009). *Mixed Effects Models and Extensions in Ecology with R.* New York, NY: Springer.

